# Policy Endorsement and Booster Shot: Exploring Politicized Determinants for Acceptance of a Third Dose of COVID-19 Vaccine in China

**DOI:** 10.3390/vaccines11020421

**Published:** 2023-02-12

**Authors:** Ruifen Zhang, Jun Yan, Hepeng Jia, Xi Luo, Qinliang Liu, Jingke Lin

**Affiliations:** 1 School of Communication, Soochow University, Suzhou 215127, China; 2 School of Journalism and Information Communication, Huazhong University of Science and Technology, Wuhan 430074, China; 3 School of Public Health, Soochow University, Suzhou 215127, China; 4 School of Journalism and Communication, Sun Yat-sen University, Guangzhou 510006, China

**Keywords:** COVID-19, vaccination, policy endorsement, nationalism, booster shot

## Abstract

China’s recent termination of strict COVID-19 control necessitates taking a booster vaccine shot as a precaution against the pandemic as quickly as possible. A large body of research has examined people’s attitudes toward and intentions for the booster shot. However, most studies failed to explore how China’s sociopolitical context has shaped their attitude regarding the booster jab take-up. The current study utilizes data from a national survey adopting quota sampling to analyze the Chinese public’s medical and non-medical considerations to determine their intention for the third dose of the COVID-19 vaccine. The study found that thanks to China’s initial successful lockdown policies, personal risk and benefit perceptions did not dominate their views regarding booster vaccination. Instead, respondents’ gender, nationalism, endorsement of the zero-COVID policy, self-efficacy regarding vaccination, and perceived infection severity were the major factors underlying their booster shot intention. The situation highlights how the politicized context of China’s COVID-19 control has impacted people’s plans to practice preventive behaviors. It is necessary to offset the negative consequences. One strategy is to educate the Chinese public with more medically relevant information to help them make rational choices regarding vaccination and other protective measures. On the other hand, such education can utilize this nationalistic mental status to enhance the persuasion effect.

## 1. Introduction

After three years of a strict zero-tolerance policy toward the severe acute respiratory syndrome coronavirus 2 (SARS-CoV-2) that caused the COVID-19 pandemic, China suddenly abandoned its zero-COVID policy in early December 2022 [1]. Meanwhile, the country began to strengthen its effort to improve the coverage of COVID-19 booster vaccines, especially among the elderly [2]. Given the real-world evidence of substantially increased protection from the booster vaccine dose against mild and severe infections [3,4], it is more than necessary to examine people’s intention to get a booster shot against COVID-19 and its determinants [5].

While a batch of studies has examined factors associated with people’s booster shot intention in China and worldwide [6,7,8,9], most have stopped at analyzing regular health considerations such as risk perceptions and perceived individual benefits. However, in China, harsh lockdowns have resulted in an extremely low incidence of new infections, causing the widespread perception of low infection susceptibility [10]. The situation may distort the results of the conventional approach of using perceived risks and benefits to predict vaccination intention. On the other hand, politicized factors have been found to drive people’s attitudes towards COVID-19 prevention and vaccinations worldwide. Whereas ideology and partisanship were regular variables used in the West to examine political determinants [11,12], in China, constructs measuring people’s recognition of the state have been identified to robustly link to their preventive behaviors and vaccination intention [13,14,15,16]. Due to the strong influence of these constructs—which were often conceptualized using nationalism—in the highly politicized COVID-19 fight in China, it is necessary to test the role of political determinants underlying Chinese people’s intention to take booster shots against COVID-19.

As with other determinants, people’s political beliefs function differently in divergent groups. Therefore, it is necessary to examine their role together with demographic information. Many studies have confirmed that demographic variables such as gender, age, education level, and income are factors that affect COVID-19 vaccination willingness [17,18,19]. We will also investigate the demographic factors linked to people’s intention to take COVID-19 booster jabs.

Although the primary goal of the current study is to examine political determinants underlying the Chinese public’s intention to receive a booster jab, it is necessary to place them into the context of people’s regular health considerations, including their risk and benefit perceptions. The Health Belief Model (HBM) is a widely used framework using the desire to avoid a negative health consequence (risk avoidance) to motivate people to take beneficial health actions (benefit perception) [20,21,22]. Several recent studies have used HBM to investigate how people’s high perceived risk (in terms of disease susceptibility and perceived severity) and their perceived benefit of getting vaccinated influenced their intention to accept booster jabs [23,24,25]. In this study, we incorporated demographic variables into an HBM model that also covers risk and benefit perception, political beliefs, and other relevant variables as determinants for people’s willingness to take the COVID-19 booster vaccine. In addition to perceived personal risks, we also tested the role of the perceived risk of being quarantined, because when the data for this study was collected, China’s mandatory COVID-19 lockdown measures wreaked public havoc.

Under HBM, self-efficacy, represented here by the confidence that one can handle vaccination-related issues, is often examined together with perceived risks, benefits, and barriers [26]. It is generally believed that the higher the self-efficacy, the more willing to take the COVID-19 vaccine [27]. Hence, we also investigated the association between self-efficacy and the willingness to take booster shots.

In the COVID-19 setting, the impact of conspiracy theories on people’s preventive behaviors and vaccination was frequently investigated. Conspiracy theories weaken the authority of scientists and doctors and have been found to reduce people’s willingness to vaccinate [28,29,30]. However, different conspiracy theories may have varying consequences. In the COVID-19 vaccine setting, we tested the two most popular types of such theories—those related to the origin of SARS-CoV-2 and those about vaccines [31]. In addition to concrete conspiracy theories, we also investigated respondents’ conspiratorial thinking, which represents a general susceptibility to explanations based on conspiracy theories.

To counter the negative impact of conspiracy theories, scholars have proposed that knowledge, both scientific knowledge in general and specific knowledge, prevents the public from being influenced by conspiracy theories [32,33]. Correspondingly, Luo and Jia [34] found that science literacy, an indicator used to assess both general scientific knowledge and analytical thinking, negatively affected respondents’ beliefs in COVID-19 conspiracy theories. Yang et al. (2021) [31] examined the role of science literacy and vaccine knowledge in people’s vaccination intention, and found that the latter knowledge played a significant role. In this study, we replicated Yang et al. (2021) [31] to examine whether and how science literacy and vaccine knowledge are associated with Chinese people’s intention to take booster shots.

On top of these factors, we investigated the role of political factors in influencing respondents’ intention to take booster shots, which was the central task of the current study. For this task, we first examine nationalism, which, as mentioned above, has been repeatedly found to be linked to people’s intention to take preventive measures and vaccination and their acceptance of mandatory vaccination policies [13,15,16,35]. Nationalism represents one’s embracing of his/her government/state. It is plausible that nationalism should also predict people’s willingness to take booster jabs, as they understand that booster vaccination is cherished by the government.

Nationalism as a construct can be understood as one’s identification with his or her national group. Indeed, national identity has been found to predict people’s support for public health measures against the COVID-19 pandemic globally [36]. However, in the COVID-19 prevention setting, people not only identify with an abstract state or government, but also stand with certain policy lines. In China, the effort to control the pandemic is highly politicized [37]. The public domain has become polarized on whether to stick to the zero-COVID policy, with those supporting the government tending to favor zero-COVID, at least before the government itself gave up on the policy [38]. Facing the situation, we designed a construct—policy endorsement—to measure people’s identification with the strong control principles underlying China’s zero-COVID policies. The plan was to examine whether people’s identification with the central policy goal in China could be associated with their booster shot intention.

## 2. Research Method and Samples

### 2.1. Research Method

The questionnaire is one of the important means to investigate public health behavior willingness and its influencing factors. This research method has been used in many previous studies on Chinese public epidemic prevention behavior [8,10,15,39]. Although it is difficult for survey researchers to obtain more profound and more nuanced ideas and understanding from participants compared with in-depth interviews, the closed-ended questionnaire has high efficiency, and the results can be processed uniformly for quantitative statistical analysis. Compared with in-depth interviews, survey research can be more representative and can, relatively speaking, avoid researchers’ and respondents’ bias. To explore how China’s sociopolitical context has shaped the public’s attitude regarding booster jab uptake, this paper adopts questionnaires as the research method and collects various dimensions of information from the subjects. The reliability of the scale used in this questionnaire has passed the reliability test (see the next section for details).

The descriptive analysis was performed to show the frequency and proportion of each variable, and the data were analyzed by multiple linear regression to test the relationship between the independent and dependent variables. The software used for statistical analysis was SPSS Version 25.0, and we set 95% as the confidence level.

### 2.2. Data Collection

In January 2022, we surveyed 1510 people through quota sampling provided by the research company “Diaoyanba” based in Shanghai. The research company’s sample database has a capacity of more than 5 million. The online sample came from all provinces in China, with the distribution of region (at the provincial level), age, and gender in line with the Statistical Yearbook 2020 of China. When implementing the project, stratified random sampling was adopted to randomly select members in the “Online Research Sample Database” according to city, gender, and age; then, the company sent point-to-point emails to selected members, inviting them to come and participate in answering questions.

The size of the number of subjects depends on what kind of statistical analysis is used. In general, in regression analysis, the ratio of the number of subjects selected to the number of items that measure independent variables is usually 10:1, and the number of simultaneous subjects should be at least 100 [40]. There are 55 items that measure 16 independent variables in this article. So, the current sample size, which is far more than ten times as large as the number of items, meets this requirement.

Due to the lack of an ethical review committee of social science institutions in China, the researchers applied to the school management of their university for approval of the research plan. Participants were informed that they could opt out at any time during the response process if they felt uncomfortable and that they would be anonymized.

### 2.3. Variable Measurement

We collected 1510 participants’ demographics (e.g., age, gender, monthly income, and education level) and kept 1472 people based on “having been vaccinated” but not “receiving booster vaccine.” At this time, the coverage of the first and second doses of the COVID-19 vaccine in China was high, but the implementation of booster shots had just begun.

Since there are only two brands of COVID-19 vaccine in China—Sinovac and Sinopharm, both of which are produced domestically—we did not take the vaccine brand as an independent variable.

In addition to demographic information, we also examined the following five aspects: (1) Intention to take COVID-19 booster shot; (2) risk and benefit perceptions (perceived susceptibility, perceived severity, perceived social threat, perceived benefit, and self-efficacy; (3) beliefs in conspiracy theories (including vaccine-related conspiracy theories and COVID-19-related conspiracy theories) and conspiratorial thinking; (4) nationalism and zero-COVID policy endorsement; (5) science literacy and vaccine knowledge.

For the independent variables involved in risk and benefit perceptions, collective beliefs and comparative thinking, nationalism, and policy endorsement, this study used five-point or seven-point scales for measurement, and the score of the independent variables was equal to the mean value of their respective questions. The specific scale is shown in the next section. The scores for science literacy and vaccine knowledge were calculated in different ways—one point was added for each correct answer, and no point was added for the wrong and “I don’t know” answers.

## 3. Results

The descriptive statistics for the variables other than the demographic variables are shown below (Table 1). In the first six sub-summaries of this section, the scale items for each of the variables involved in the study are given, along with the proportion of the distribution.

### 3.1. Demographic Characteristics

Among all participants, males accounted for about 51.2%, and young and middle-aged people accounted for a relatively high proportion (18–29, 33.4%; 30–39, 33.8%); participants had a higher-education degree (bachelor’s degree, 35.3%; junior college, 25.4%). Participants mostly had a monthly income of less than RMB 5000 (RMB 3001–5000, 39.1%; RMB 3000 or less, 25.1%). See Table 2 for details. There was a lack of people over 60 years of age in the collected samples. The response rate for the elderly’s participation in questionnaires is normally much lower due to their unfamiliarity with the Internet. Although COVID-19 poses a great threat to the health of the elderly, the purpose of this study was not to focus on this group of people, but to focus on the willingness of all age groups to take booster vaccines and its influencing factors. Therefore, we believe that the lack of people over 60 years of age did not have a significant impact on the research results.

### 3.2. Intention to Take the Booster Shot

Two questions measuring participants’ willingness to receive a COVID-19 booster shot had good internal consistency (Cronbach’s α = 0.96). A seven-level scale measured these two questions. The data showed that the participants were willing to receive booster doses. On average, participants who wanted to (including “Slightly Agree”, “Agree” and “Totally Agree”) (M = 81.75%) were much more numerous than participants who resisted receiving a booster dose (including “Totally Disagree”, “Disagree” and “Slightly Disagree”) (M = 7.3%). See Table 3 for details.

### 3.3. Risk and Benefit Perception

Next, we measured participants’ perceived susceptibility, perceived severity, perceived social threat, and perceived benefit of vaccination with four questions, as shown in Table 4. Overall, the participants had a low level of perceived susceptibility, with only 9.5% believing that they were at high risk of infection; 65.9% were not worried about being quarantined or isolated by others due to COVID-19, but they had a higher level of perceived severity (75.4%).

We tested participants’ self-efficacy through four questions (Cronbach’s α = 0.79), which showed that participants had a high perception of self-efficacy for their COVID-19 vaccination (M = 4.15, SD = 0.8) (Table 5).

### 3.4. Conspiracy Theories and Conspiratorial Thinking

We investigated two types of conspiracy theories: vaccine-related conspiracy theories (Cronbach’s α = 0.89) and COVID-19-related conspiracy theories (Cronbach’s α = 0.72). The former contained five items, and the latter contained six items. They were all selected from the conspiracy theories that prevailed in the real world, as detailed in Table 6. We had repeatedly tested these theories in previous surveys. We examined participants’ trust in conspiracy theories on five levels. The results showed that participants had a lower level of trust in vaccine conspiracy theories, and on average, those who held “Totally Disagree” (M = 37.2%) accounted for the largest proportion of attitudes, followed by “Neutral” (M = 29.4%) and “Disagree” (M = 19.9%). For the COVID-19 conspiracy theory attitude, the largest proportion of participants was “Neutral” (M = 32.4%), followed by “Totally Disagree” (M = 31.9%)**.**

In addition, we tested participants’ conspiratorial thinking, a dimension consisting of five questions (Cronbach’s α = 0.89). It measured people’s tendency to accept the incomprehensibility of governments or elites’ behaviors. Its reliability has been tested by previous studies [41,42].

Overall, the participants had modest beliefs in both vaccine conspiracy theories (M = 2.27, SD = 1.0) and COVID-19 conspiracy theories (M = 2.60, SD = 0.77). However, they had high conspiratorial thinking (M = 3.23, SD = 1.0).

### 3.5. Nationalism and Zero-COVID Policy Endorsement

We selected six questions(Table 7) based on an established measure to examine nationalism [43]. Previous studies have repeatedly adopted the measure [13,44]. Those questions had good internal consistency (Cronbach’s α = 0.83). The respondents had a high level of nationalism (M = 5.98, SD = 1.04).

The following four questions (Table 8) measured the degree of public acceptance of zero-COVID policies at the time the questionnaire was released, with good consistency (Cronbach’s α = 0.73). The data show that the proportion of the public with a favorable attitude is much higher than that with an opposing attitude: Totally Agree (M = 42.4%), Agree (M = 28.5%); Disagree (M = 5.0%), Totally Disagree (M = 3.7%).

### 3.6. Science Literacy and Vaccine Knowledge

We measured participants’ science literacy using 11 yes/no and multiple-choice questions to examine participants’ familiarity with scientific procedures, analytical thinking, and basic scientific knowledge, respectively. The options in bold in the Table 9 below are correct. The questions were adopted from a well-established, globally used instrument [45]. One point was added for each correct answer, and no point was added for the wrong and “I don’t know” answers. The data showed that the participants had an average level of science literacy (M = 5.7, SD = 2.68).

We also measured participants’ vaccine knowledge using six yes/no questions (Table 10). The questions were adopted from Yang et al. [31]. One point was added for each correct answer, and no point was added for the other answers. The options in bold in the table below are correct, and it can be seen that except for a few questions, the correct rate is low, and the public’s vaccine knowledge is at an upper middle level (M = 3.80, SD = 1.25).

### 3.7. The Influencing Factors of Intention to Take Booster Shot

Finally, a multiple linear regression model was used to detect the relationship between demographic variables, risk and benefit perception, self-efficacy, conspiracy theory beliefs, science literacy and vaccine knowledge, nationalism and policy endorsement, and booster shots intention (Table 11). The adjusted R^2^ of this model is 0.132. 

The first model focused on the role of demographic variables and found that individual characteristics were not nearly related to participants’ willingness to receive booster shots. Only gender was associated with the willingness to receive boosters—women were more likely than men to receive booster doses. The significance of this factor continued until the fifth model (*β* = 0.065, *p* < 0.01).

In the second model, we added the classic variables linked to the Health Belief Model, and added the independent variable “perceived social threat” according to the relevant pandemic control situation. We found that perceived severity and perceived benefit had a significant effect on booster vaccination, but in Model 5, the significant influence of perceived benefit eventually disappeared as nationalism and policy endorsement were added. Perceived severity (*β* = 0.087, *p* < 0.01) positively impacted the intention for booster vaccination. In addition, self-efficacy significantly affected the willingness to be vaccinated with the booster shot (*β* = 0.145, *p* < 0.000).

The third model showed the influence of beliefs in conspiracy theories and conspiratorial thinking. Conspiratorial thinking and vaccine-related conspiracy beliefs did not work at all, and the influence of COVID-19-related conspiracy beliefs disappeared with the addition of nationalism and policy endorsement.

In the fourth model, we measured the role of scientific literacy and vaccine knowledge. Both had no effect on the willingness to get a booster shot.

In the fifth model, nationalism (*β* = 0.078, *p* < 0.01) and policy endorsement (*β* = 0.186, *p* < 0.000) had a significant positive influence. It showed that among the factors influencing the willingness to be vaccinated, gender, self-efficacy, perceived threat severity, nationalism, and policy endorsement had a significant impact on the willingness to take a booster jab. 

## 4. Discussions

The findings offered fresh evidence for the unique role that the Chinese public’s political beliefs have played in influencing their intention to take a booster shot against COVID-19. Political beliefs—nationalism and policy endorsement—and determinants related to these beliefs, such as efficacy and perceived severity of COVID-19 infection, have played dominant roles in our studies. This study differed from previous studies [5,17] in that, among the investigated demographic variables (age, education level, gender, and income), only gender played a minor predictive role.

Factors normally covered in the Health Belief Model—perceived susceptibility of infection, perceived seriousness, perceived benefits, and self-efficacy—in Model 2 significantly increased the explanatory power. The perception of the severity of infection and self-efficacy were significantly associated with booster shot intention. However, perceived susceptibility was not a statistically significant determinant, possibly because when this questionnaire was answered (January 2022), China was successfully implementing its zero-COVID measures, making the ordinary public perceive a low infection risk.

One surprising finding is the negative prediction of the perceived benefit of vaccination against COVID-19. Both the classical HBM model and recent studies on booster shot intention indicate that the perceived benefits should be one of the primary drivers for people to take the shot [5,17,46,47]. However, in our study, the more benefits people could perceive from taking such a shot, the more likely they were to not take the jab. The significance only disappeared when nationalism and policy endorsement entered the model in step 5. One possible explanation is that the perceived benefit that we tested covered the overall advantages of vaccination (preventing COVID-19 infection) rather than just booster shots, while our investigated respondents had received basic COVID-19 vaccines but not the booster shot. In an environment with low infection risk, people who have a stronger personal benefit perception of vaccination might be those who believe in the effectiveness of the basic COVID-19 vaccines they have already taken and have a lower consciousness of getting additional ones. This point can again be linked to their confidence in China’s powerful epidemic control capacity and its political system that ensured the fast production and delivery of COVID-19 vaccines.

Models 3 and 4 added conspiracy-theory-related and knowledge-related determinants, but the two models did not remarkably expand the explanatory scope, and their addition of variances was not significant either. The results indicated that conspiracy beliefs and conspiratorial thinking, as well as science literacy and vaccine-specific knowledge, did not function well in influencing our respondents’ intention to take booster shots. The results were different from previous studies that found that vaccine-related knowledge was the primary determinant for COVID-19 vaccination [6,17,31]. This difference could be caused by our fully vaccinated sample. For them, vaccine knowledge and science literacy did not add additional impetus for booster shot uptake. 

The final model significantly increased the explained variance of booster shot intention. Both nationalism and policy endorsement were positively associated with the intention. Meanwhile, another two statistically significant determinants—perceived severity of infection and self-efficacy—had lowered coefficients, indicating that their predictive roles were shared by nationalism and policy endorsement. Overall, zero-COVID policy endorsement and self-efficacy were the most robust determinants. Taken together, it was not the individually perceived infection risk or the perceived vaccination benefits, but primarily the agreement to the zero-COVID policies and identity to the powerful state that dominantly drove the Chinese public to take a booster shot. In the scenario, self-efficacy, or confidence that one can easily get vaccinated and vaccination-related support, was not just a matter of individualistic self-confidence, but also an assessment of government or state empowerment for them to take the vaccine.

Overall, our study revealed a nationalistically shaped mental world underlying our respondents’ intention to take a booster shot against COVID-19. This is consistent with several recent studies highlighting the role of nationalism/loyalty to the state in shaping Chinese respondents’ preventive behaviors against the pandemic [14,15,16,35]. To a certain degree, it is also consistent with findings on the role of cultural cognition in predicting policy compliance [48,49].

## 5. Theoretical Contribution and Practical Implications

The main theoretical contribution of this paper is that it reveals an alternative landscape regarding the determinants of people’s willingness to vaccinate. This study expanded the existing body of literature that explores the politicization of the Chinese public’s attitude towards and intention for COVID-19 vaccination [13,16,35,39]. The second contribution is that it shows that the influence of the policy itself can affect the public attitudes.

The results are also practically significant. Although, according to our study, it is the politicized factors rather than personal risk and benefit perception that dominate the public’s intention for vaccination, we cannot ignore personal risks and benefits. However, the message encouraging people to evaluate the trade-off between personal risks and benefits can be promoted in a nationalistic tone to improve its persuasiveness and effectiveness [15].

Finally, our research has some limitations that are worth addressing for further improvement. First, this study was based on a cross-sectional survey, which cannot ensure a causational conclusion.

Second, as mentioned above, our sample lacked people over 60 years of age. We have explained before that the insufficiency should not discount the validity of our overall conclusions; however, it is certain that more diversified age groups will bring new observable dimensions.

Third, our January 2022 data are relatively old, especially considering that people’s risk perceptions might have been dramatically changed after China abandoned its zero-COVID policy in early December 2022. However, the role of the strongest determinants—nationalism and people’s policy endorsement—should not have changed in a new period.

Fourth, the influencing factors on booster vaccination measured in this paper were not comprehensive. For example, we lacked the statistics on participants’ marital status and the measurement of the effect of vaccine brands. However, we do not think that this affected the results of this paper, because our paper mainly focused on exploring how China’s sociopolitical context has shaped the public’s attitude regarding the booster jab uptake. Concerning the vaccine brand, as at the time of our survey, there were only two brands—Sinovac and Sinopharm, both inactivated—available in China, the brand issue was not very relevant; although, with the approval of more and more brands of COVID-19 vaccines in China, it should be necessary in future studies to examine the impact of vaccine brand on people’s vaccination intention.

In general, the above limitations do not negate the validity of this study. To solve the limitations, follow-up research can use updated data or more diversified methods to explore the political aspects of Chinese people’s health attitudes and behaviors.

## Figures and Tables

**Table 1:** Descriptive statistics and units of the continuous variables.

Variables	Minimum	Maximum	Mean	SD
Booster vaccination intention	1 (=Totally Disagree)	7 (=Totally Agree)	5.9939	1.55369
Perceptions of susceptibility	1 (=Totally Disagree)	5 (=Totally Agree)	1.94	1.186
Perceptions of severity	1 (=Totally Disagree)	5 (=Totally Agree)	4.04	1.253
Perceptions of being quarantined	1 (=Totally Disagree)	5 (=Totally Agree)	2.90	1.394
Perceptions of benefits	1 (=Totally Disagree)	5 (=Totally Agree)	2.49	0.842
Self-efficiency	1 (=Totally Disagree)	5 (=Totally Agree)	4.1559	0.86434
Vaccine-Related conspiracy beliefs	1 (=Totally Disagree)	5 (=Totally Agree)	2.2656	1.00612
COVID-19-related conspiracy beliefs	1 (=Totally Disagree)	4 (=Totally Agree)	2.5974	0.77228
Conspiratorial thinking	1 (=Totally Disagree)	5 (=Totally Agree)	3.2313	1.04413
Science literacy	0 (=Correct Number)	11 (=Correct Number)	5.7357	2.68315
Vaccine knowledge	1 (=Correct Number)	6 (=Correct Number)	3.7985	1.25141
Policy obedience	1 (=Totally Disagree)	5 (=Totally Agree)	4.0093	0.79135
Nationalism	1 (=Totally Disagree)	7 (=Totally Agree)	5.9865	1.04636

**Table 2:** Participants’ demographic characteristics (*n* =1472).

Variable	% (*n*)
Age	
18–29	33.4 (492)
30–39	33.8 (497)
40–49	22.4 (330)
50–59	10.4 (153)
Gender	
Male	51.2 (753)
Female	48.8 (719)
Education level	
Junior high school and below	14.1 (208)
Senior high school	23.2 (342)
Junior college	25.4 (374)
Bachelor degree	35.3 (519)
Postgraduate and above	2.0 (29)
Monthly income (RMB)	
3000 or less	25.1 (370)
3001–5000	39.1 (575)
5001–10,000	20.4 (300)
10,001–20,000	10.3(151)
More than 20,000	5.2 (76)

**Table 3:** Intention to take booster shots (*n* = 1472).

Intention to Booster Shots	Totally Disagree	Disagree	Slightly Disagree	Neutral	Slightly Agree	Agree	Totally Agree
I am likely to get a COVID-19 booster shot (the third dose) recently.	4.8 (71)	1.3 (19)	1.3 (19)	11.3 (166)	6.1 (90)	17.1 (252)	58.1 (855)
I am planning to get a COVID-19 booster shot (the third dose) recently.	4.0 (59)	1.0 (15)	1.2 (17)	11.5 (170)	6.3 (93)	16.4 (242)	59.5 (876)

**Table 4:** Participants’ benefit and risk perceptions (*n* = 1472).

Participants’ Benefit and Risk Perceptions	Totally Disagree	Disagree	Neutral	Agree	Totally Agree
I may have COVID-19 for a good chance.	52.0 (766)	18.0 (265)	20.4 (300)	3.5 (52)	6.0 (89)
COVID-19 is a major threat to my health.	8.1 (119)	5.7 (84)	10.9 (160)	24.9 (366)	50.5 (743)
I am worried that I will be quarantined or isolated because of getting COVID-19.	22.1 (325)	18.3 (270)	25.5 (376)	15.8 (233)	18.2 (268)
Getting vaccinated keeps me from COVID-19.	13.9 (204)	30.2 (444)	50.8 (748)	3.2 (47)	2.0 (29)

**Table 5:** Participants’ self-efficacy (*n* = 1472).

Is the Following Statements about COVID Vaccines Relevant to You?	Totally Disagree	Disagree	Neutral	Agree	Totally Agree
I can easily get vaccinated.	4.3 (63)	3.9 (57)	15.7 (231)	14.9 (219)	61.3 (902)
I can judge the pros and cons of the covid vaccine.	4.3 (63)	2.9 (42)	21.9 (323)	20.5 (302)	50.4 (742)
I can make the decision to get vaccinated very easily.	2.2 (33)	2.1 (31)	14.1 (207)	16.9 (249)	64.7 (952)
I can find an expert or knowledgeable person to consult about COVID vaccination.	5.8 (85)	6.7 (99)	24.4 (359)	20.2 (297)	42.9 (632)

**Table 6:** Participants’ belief in conspiracy theories and conspiratorial thinking (*n* = 1472).

Belief in Vaccine-Related Conspiracy Theories	Totally Disagree	Disagree	Neutral	Agree	Totally Agree
Pharmaceutical companies often falsify vaccine effectiveness data.	43.5 (640)	17.5 (258)	25.1 (370)	4.6 (68)	9.2 (136)
Governments often cover up vaccine safety deficiencies.	32.6 (480)	20.7 (305)	31.7 (467)	7.1 (105)	7.8 (115)
Pharmaceutical companies conceal vaccines’ dangers.	30.0 (441)	20.2 (297)	35.3 (520)	6.8 (100)	7.7 (114)
The public was blinded on the effectiveness of vaccines.	33.2 (489)	18.3 (269)	32.1 (472)	8.8 (130)	7.6 (112)
The fact that vaccinating children is harmful has been deliberately concealed.	46.7 (688)	22.8 (335)	22.8 (335)	3.4 (50)	4.3 (64)
Belief in COVID-19-related Conspiracy theories	Totally Disagree	Disagree	Neutral	Agree	Totally Agree
COVID-19 virus flowed from foreign military laboratories.	16.4 (242)	11.1 (164)	41.4 (610)	13.5 (198)	17.5 (258)
A virus research institute in Wuhan, China, is the origin of the COVID-19 virus.	57.1 (841)	16.8 (247)	24.9 (367)	0.7 (10)	0.5 (7)
Foreign athletes who participated in the Wuhan Military Games were infected with COVID-19, which led to the spread of the pandemic in China.	18.3 (269)	12.0 (177)	37.6 (553)	14.1 (207)	18.1 (266)
5G technology promotes the spread of COVID-19.	60.9 (897)	14.5 (214)	17.3 (254)	3.4 (50)	3.9 (57)
The genetic sequence of COVID-19 indicates that it is synthetic.	26.4 (389)	13.1 (193)	41.0 (604)	9.2 (136)	10.2 (150)
The earlier emergence of COVID-19 type circulating in the US indicates that the US is more likely to be the source of the virus.	12.1 (178)	7.7 (114)	32.1 (473)	20.8 (306)	27.2 (401)
Conspiratorial Thinking	Totally Disagree	Disagree	Neutral	Agree	Totally Agree
The public doesn’t know the truth about significant things.	10.9 (161)	8.6 (127)	34.8 (512)	17.1 (251)	18.6 (421)
We don’t understand the reasons behind the official decision.	12.0 (176)	11.5 (170)	37.0 (545)	17.1 (251)	22.4 (330)
The public is under government surveillance.	18.4 (271)	16.8 (248)	37.9 (558)	11.8 (173)	15.1 (222)
Many seemingly unconnected events are often the result of secret activities.	11.5 (169)	9.1 (134)	38.9 (573)	19.8 (291)	20.7 (305)
Political decisions are influenced by some secret organizations.	12.4 (183)	9.0 (280)	38.1 (561)	19.0 (280)	21.5 (316)

**Table 7:** Nationalism score of participants (*n* = 1472).

Nationalism	Totally Disagree	Disagree	Slightly Disagree	Neutral	Slightly Agree	Agree	Totally Agree
I would prefer to be a Chinese citizen than any other country.	1.6 (23)	0.4 (6)	1.8 (26)	6.0 (89)	3.9 (58)	10.0 (147)	76.3 (1123)
My country is better than most others.	0.7 (10)	1.0 (14)	2.0 (29)	6.4 (94)	5.1 (75)	8.9 (131)	76.0 (1119)
When the government makes mistakes, we should also support them.	22.4 (329)	10.4 (153)	42.9 (149)	21.0 (309)	7.8 (115)	8.6 (126)	19.8 (291)
I felt great when I saw the Chinese national flag flying.	0.5 (8)	1.0 (14)	1.6 (24)	9.7 (143)	5.4 (79)	6.5 (96)	75.3 (1108)
The fact that I am Chinese is an important part of who I am.	0.9 (13)	0.8 (12)	4.0 (59)	8.5 (125)	5.0 (73)	7.1 (105)	73.7 (1085)
I am proud as a Chinese.	0.6 (9)	0.1 (2)	1.4 (21)	9.9 (21)	4.8 (71)	6.6 (97)	76.5 (1126)

**Table 8:** Participants’ zero-COVID policy endorsement (*n* = 1472).

Acceptance of Zero-COVID Policies	Totally Disagree	Disagree	Neutral	Agree	Totally Agree
As long as there is a close connection in a place, nucleic acid testing should be carried out within a certain range.	3.2 (47)	3.3 (49)	12.2 (179)	27.1 (399)	54.2 (47)
Anyone who comes from abroad should take a nucleic acid test.	4.6 (67)	6.0 (88)	18.4 (271)	28.2 (415)	42.9 (631)
People from foreign countries should be quarantined for more than 1 month.	3.5 (47)	7.0 (103)	27.4 (404)	29.5 (434)	32.5 (479)
If the COVID-19 infection is not completely cleared, there will be an uncontrollable public health crisis.	3.3 (49)	3.5 (52)	24.3 (357)	29.0 (427)	39.9 (587)

**Table 9:** Participants’ science literacy (*n* = 1472).

Science Literacy			
Two scientists wanted to know if a drug to treat high blood pressure was effective. The first scientist gave the drug to 1000 patients with high blood pressure and then observed how many patients had a decrease in blood pressure; The second scientist divided the patients into two groups, the first group of 500 hypertensive patients taking the drug and the other group of 500 patients not taking the drug, to see how the blood pressure dropped in the two groups. Which of the first and second scientists tested the drug’s effect more effectively?	first	second	Don’t Know
	10.6 (156)	78.7 (1159)	10.7 (157)
Doctors tell a couple that because they share the same pathological genes if they have a child, the chance of that child developing a genetic disease is 1 in 4. The following statement, whether you think it is correct or not.	false	true	Don’t Know
If they have three children, none of the three children will get a genetic disease.	64.3 (947)	5.6 (83)	30.0 (442)
If their first child has a genetic disease, the next three children will not have it.	57.0 (839)	10.3 (151)	32.7 (482)
If the first three children are healthy, then the fourth child must have a genetic disease.	54.1 (797)	11.6 (171)	34.2 (504)
Their children can all get genetic diseases.	8.8 (130)	66.6(981)	24.5 (361)
Do you think the following statement is correct?	false	true	Don’t Know
Coronaviruses can cause SARS and pneumonia but not colds.	59.0 (869)	8.4 (123)	32.6 (480)
Electrons are smaller than atoms.	24.6 (362)	15.5 (522)	39.9 (588)
The mother’s genes determine whether the fetus is a boy or a girl.	81.5 (1200)	5.1 (75)	13.4 (197)
Lasers are produced by converging sound waves.	24.0 (354)	20.6 (303)	55.4 (815)
Antibiotics (such as penicillin, streptomycin, or cephalosporin) kill both bacteria and viruses.	47.4 (698)	22.3 (328)	30.3 (446)
If genetically modified fruits are eaten, a person’s genes may change.	55.9 (823)	10.9 (161)	33.2 (488)

**Table 10:** Participants’ vaccine knowledge (*n* = 1472).

Vaccine Knowledge	False	True	Don’t Know
The disease can always be cured, so vaccine is not necessary.	81.6 (1201)	2.6 (38)	15.8 (233)
Smallpox would not have been eradicated without widespread vaccine application.	3.6 (53)	78.9 (1161)	17.5 (258)
Premature vaccination will affect the normal development of children’s immune system.	42.9 (631)	14.8 (218)	42.3 (623)
Vaccination does not increase the occurrence of allergies.	35.5 (522)	18.5 (272)	46.1 (678)
If children are not given as many vaccines, they will be more resistant to the disease.	60.3 (887)	9.3 (137)	30.4 (448)
Vaccination can trigger diseases such as autism, multiple sclerosis, and diabetes.	45.9 (675)	10.7 (157)	43.5 (640)

**Table 11:** Factors associated with the intention of taking booster shots (*n* = 1472).

Variable	Intention of Taking Booster Shots				
	Model 1	Model 2	Model 3	Model 4	Model 5
	*β*	*β*	*β*	*β*	*β*
Demography					
Gender (ref. male) Female	0.084 **	0.069 **	0.073 **	0.074 **	0.065 *
Age	0.017	0.014	0.011	0.011	0.024
Education (ref. Junior high school and below)					
Junior high school	−0.080	−0.078	−0.076	−0.074	−0.063
Junior college	0.040	0.021	0.021	0.021	0.024
Undergraduate degree	−0.007	−0.005	−0.002	0.004	0.026
Postgraduate degree	0.017	0.028	0.032	0.033	0.041
Income	−0.036	−0.037	−0.037	−0.035	−0.039
Risk and Benefit Perceptions					
Perceptions of susceptibility		−0.016	−0.013	−0.013	−0.013
Perceptions of severity		0.148 ***	0.141 ***	0.145 ***	0.087 **
Perceptions of being quarantined		0.003	0.003	0.006	0.001
Perceptions of benefits		−0.078 ***	−0.076 **	−0.076 **	−0.022
Self-efficiency		0.213 ***	0.214 ***	0.211 ***	0.145 ***
Conspiracy beliefs and Conspiratorial Thinking					
Vaccine-related conspiracy beliefs			−0.034	−0.036	0.002
COVID-19-related conspiracy beliefs			0.073 *	0.074 *	0.041
Conspiratorial thinking			0.002	0.003	−0.004
Literacy and Knowledge					
Science literacy				−0.033	−0.035
Vaccine Knowledge				0.048	0.056
Nationalism and Policy endorsement					
Nationalism					0.078 **
Zero-COVID policy endorsement					0.186 ***
Model statistics					
Adjusted *R*^2^	0.011	0.099	0.100	0.101	0.132
Δ*R*^2^	0.017	0.091	0.003	0.003	0.032
Δ*F*	2.986 **	24.124 ***	1.489	1.692	22.460 ***

* *p* < 0.05. ** *p* < 0.01. *** *p* < 0.000.

## Data Availability

Materials and anonymous data are available from the authors by request.
